# Serine Deamination Is a New Acid Tolerance Mechanism Observed in Uropathogenic Escherichia coli

**DOI:** 10.1128/mbio.02963-22

**Published:** 2022-12-05

**Authors:** Michelle A. Wiebe, John R. Brannon, Bradley D. Steiner, Adebisi Bamidele, Alexandra C. Schrimpe-Rutledge, Simona G. Codreanu, Stacy D. Sherrod, John A. McLean, Maria Hadjifrangiskou

**Affiliations:** a Vanderbilt University, Nashville, Tennessee, USA; b Department of Pathology, Microbiology & Immunology, Vanderbilt University Medical Center, Nashville, Tennessee, USA; c Department of Urology, Vanderbilt University Medical Center, Nashville, Tennessee, USA; d Institute for Infection, Immunology & Inflammation, Vanderbilt University Medical Center, Nashville, Tennessee, USA; e Center for Innovative Technologies, Department of Chemistry, Vanderbilt University, Nashville, Tennessee, USA; University of Alberta; Washington University School of Medicine

**Keywords:** *Escherichia coli*, acid stress, bacterial stress response, metabolomics, serine

## Abstract

Escherichia coli associates with humans early in life and can occupy several body niches either as a commensal in the gut and vagina, or as a pathogen in the urinary tract. As such, E. coli has an arsenal of acid response mechanisms that allow it to withstand the different levels of acid stress encountered within and outside the host. Here, we report the discovery of an additional acid response mechanism that involves the deamination of l-serine to pyruvate by the conserved l-serine deaminases SdaA and SdaB. l-serine is the first amino acid to be imported in E. coli during growth in laboratory media. However, there remains a lack in knowledge as to how l-serine is utilized. Using a uropathogenic strain of E. coli, UTI89, we show that in acidified media, l-serine is brought into the cell via the SdaC transporter. We further demonstrate that deletion of the l-serine deaminases SdaA and SdaB renders E. coli susceptible to acid stress, similar to other acid stress deletion mutants. The pyruvate produced by l-serine deamination activates the pyruvate sensor BtsS, which in concert with the noncognate response regulator YpdB upregulates the putative transporter YhjX. Based on these observations, we propose that l-serine deamination constitutes another acid response mechanism in E. coli.

## INTRODUCTION

Acid stress is a substantial challenge to bacterial life. Acidic conditions can damage the bacterial cell envelope, rendering membrane-embedded proteins and the proton motive force established across the membrane dysfunctional or nonfunctional (1). Acidic conditions in the bacterial cell interior disturb vital physiological processes, such as enzymatic activity, protein folding, membrane, and DNA maintenance, all of which are needed for cellular function and can cause bacterial death. As a result, bacteria are equipped to withstand acidic conditions. One of the model bacterial organisms, Escherichia coli, occupies numerous environmental and host niches and encounters a range of acidic conditions in a niche-dependent manner. For example, all E. coli harbored in the gut are thought to be acquired via ingestion (2), indicating that E. coli must be able to survive the low pH of the stomach, which ranges from pH 1.5 to 3.5 (3–5). It is therefore not surprising that five acid resistance (AR) mechanisms, termed AR1 to AR5, have been identified in E. coli, all of which have been shown to be active in the gut (6, 7).

The most well characterized AR mechanisms, AR2-5, depend on the import and subsequent decarboxylation of specific amino acids. The decarboxylation reaction consumes one proton, thereby increasing the cytoplasmic pH. AR2, which has been shown to be the most effective AR mechanism in E. coli, involves the deamination of glutamine to glutamate, which is then decarboxylated to form γ-amino butyric acid (GABA) (3, 8–10). AR3 involves the decarboxylation of arginine to produce agmatine, while AR4 leads to decarboxylation of lysine to produce cadaverine. Finally, ornithine decarboxylation to putrescine is the basis of AR5 (10). The AR1 mechanism of action is not well understood.

While decarboxylation of amino acids appears to be the primary means of acid resistance in E. coli, there is evidence that deamination of amino acids can also serve to increase the intracellular pH. This is evident in AR2, where glutamine deamination not only provides the glutamate that is then decarboxylated (AR2_Q) (11, 12) but also produces ammonia which consumes a proton and diffuses out of the cell as ammonium (11, 13). Here, we demonstrate that the deamination of l-serine is an additional acid response mechanism in E. coli.

Notably, l-serine is the first amino acid consumed by E. coli when grown in complex media (14). Under aerobic conditions, it is known that l-serine is deaminated by SdaA and SdaB to produce pyruvate and ammonia (15). However, to date no metabolic role for serine deamination in E. coli has been described (16), as most of the pyruvate derived carbon is subsequently excreted from the cell (14). In Klebsiella aerogenes and Streptococcus pyogenes serine deamination has been shown to be important for regulating nitrogen balance and maintaining extracellular pH (17, 18), but the full import of serine deamination in these pathogens remains unclear.

Uropathogenic E. coli (UPEC) is an extraintestinal pathotype of E. coli, responsible for over 75% of reported urinary tract infections (19). UPEC strains have developed strategies to persist for years in the host (20–22), colonizing the gut, the vaginal space, and the bladder in asymptomatic reservoirs for long periods of time (23). UPEC has been reported to persist within the acidic environment of the vagina (pH 3.8 to 5) (24, 25) before ascending to and colonizing the urinary tract (pH 5.5 to 7) (4, 5). No studies have elucidated the mechanisms of acid tolerance in UPEC. In this work, we demonstrate that l-serine deamination is a previously unrecognized acid tolerance mechanism in UPEC. We show that under acidic conditions, serine is transported into the cell by the SdaC transporter and deaminated by the l-serine deaminases SdaA and SdaB, a process that imparts E. coli with protection from acid.

## RESULTS

### *YhjX* is upregulated in response to low pH in a manner that depends on the noncognate two-component system BtsS and YpdB.

**FIG 1:**
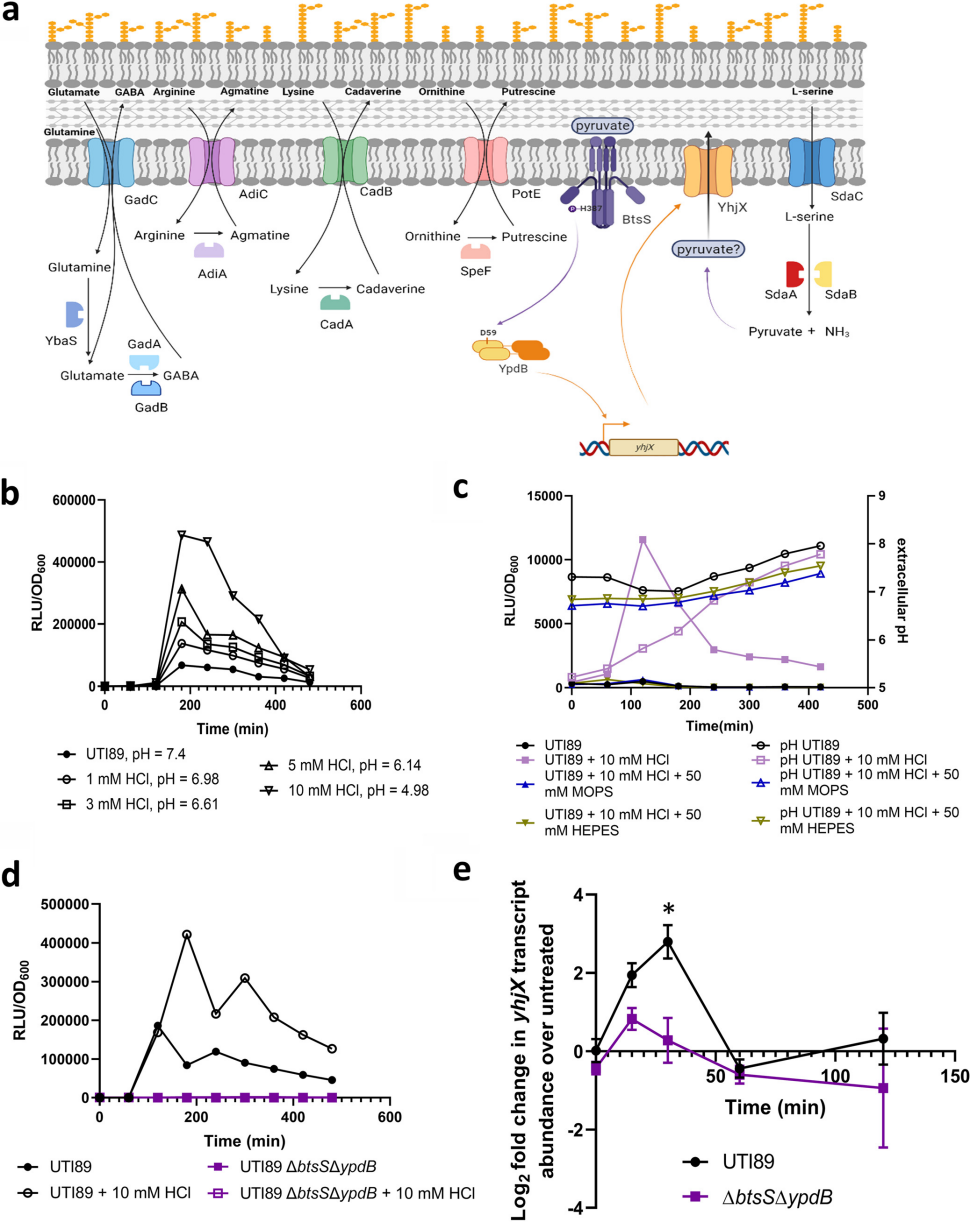
*yhjX* is upregulated in response to low pH in a manner that depends on the noncognate two-component system BtsS and YpdB. (a) Cartoon depicts currently known acid tolerance mechanisms in E. coli. Also depicted is our proposed novel acid tolerance mechanism that depends on l-serine import and deamination. The signaling system BtsS-YpdB and its downstream target *yhjX* that codes for a putative transporter, are also induced during acid stress. Cartoon was created using BioRender.com. (b–c) Graphs depict relative luminescence units (RLU) normalized to growth (OD_600_) over time, of UPEC strain UTI89 harboring the P*yhjX*-*lux* reporter in increasing concentration of HCl (b) or during growth in LB buffered to a pH of 7 with either 50 mM MOPS or 50 mM HEPES (c). (d) Luciferase reporter assay of UPEC strain UTI89 the isogenic Δ*btsS*Δ*ypdB* strain grown in the presence (initial pH of 5.0) or absence (initial pH of 7.4) of HCl. Graphs are representative of 3 independent biological repeats. (e) RT-qPCR analysis of *yhjX* transcript abundance after acid stimulation (pH of 5) in wild-type UTI89 (black) and Δ*btsS*Δ*ypdB* (purple) strains. Relative fold change was determined by the ΔΔ*C_T_* method, where transcript abundances were normalized to *gyrB* housekeeping gene transcripts. *, *P* = 0.0165 calculated by two-way ANOVA with Sidak’s multiple-comparison test. Error bars indicate SEM of three biological replicates.

Previous work indicated that l-serine induces the activation of the BtsS pyruvate sensor kinase (26), presumably due to production of pyruvate via SdaA/B mediated l-serine deamination. BtsS has been shown to induce the upregulation of the uncharacterized gene *yhjX*, in concert with a noncognate response regulator partner, YpdB (27). Interestingly, natural induction of *yhjX* has been previously reported by the Jung group to occur in *E coli* culture during midlogarithmic growth phase (28), when bacterial cell density is high and nutrient depletion of LB begins to occur. Moreover, previous studies have shown that *yhjX* is among the genes upregulated in response to acidic pH in K-12 E. coli (29). To confirm that these previous observations hold true in UPEC, we first assessed induction of *yhjX* in cystitis strain UTI89 (20). UTI89 is a sequence type ST95 strain, isolated from a patient with cystitis (20). UTI89 is an O18:K1:H7 serotype, typical of UPEC strains and its genome has been sequenced (20, 30). To monitor whether *yhjX* induction is indeed acid-responsive in UPEC, we used a previously constructed strain UTI89/P*yhjX*::lux (27) that harbors a plasmid containing the *yhjX* promoter fused to the *luxCDABE* operon (28). Luminescence and bacterial growth were monitored over time in unbuffered lysogeny broth (LB) with shaking. Cultures inoculated at near-neutral pH 7.4 to 7.6 (Fig. 1c, right y-axis) showed a peak in *yhjX* promoter activity at 180 min, coincident with late logarithmic phase of growth (Fig. 1b, Fig. S1, Data set S1). Addition of increasing concentrations of HCl to the media led to an increase in *yhjX* promoter activity that was proportional to the drop in pH (Fig. 1b and Data set S1). The addition of HCl under the conditions tested did not affect bacterial growth (Fig. S1). Addition of buffer—either MOPS or HEPES—to the acidified culture media restored neutral pH and suppressed *yhjX* promoter activity to levels observed when grown in LB with pH 7.4 (Fig. 1c and Data set S1). These results indicate that *yhjX* is indeed an acid-induced target in UPEC and corroborate previous observations in K-12 strains of E. coli.

10.1128/mbio.02963-22.1 FIG S1 Growth of strains depicted in Fig. 1, under acidic conditions. OD_600_ was measured over time of UTI89 cultures in the presence of increasing concentrations of HCl during growth in LB. Growth curves are representative of 3 biological replicates. Download FIG S1, TIF file, 0.3 MB. Copyright © 2022 Wiebe et al. 2022 Wiebe et al. https://creativecommons.org/licenses/by/4.0/ This content is distributed under the terms of the Creative Commons Attribution 4.0 International license.

The *yhjX* gene encodes a putative pyruvate transporter of the major facilitator superfamily that has not yet been shown to import or export pyruvate (28, 31). Studies have shown that pyruvate increases in the extracellular milieu as the bacterial culture reaches late exponential growth (28), and that this pyruvate is directly sensed by the BtsS histidine kinase, leading to subsequent upregulation of *yhjX* via the action of the YpdB response regulator (27). To determine whether acid-mediated induction of *yhjX* is dependent on BtsS and YpdB, a mutant lacking both *btsS* and *ypdB* (Δ*btsS*Δ*ypdB* [27]) was tested via our luminescence reporter assay in acidic and neutral conditions. These experiments showed no induction of *yhjX* in the Δ*btsS*Δ*ypdB* strain, regardless of pH in the culture media (Fig. 1d and Data set S1). To validate these findings, *yhjX* steady-state transcript over time was also monitored by RT-qPCR and TaqMan based chemistry in acidified and nonacidified cultures of wild-type UTI89 and the isogenic Δ*btsS*Δ*ypdB* mutant. Transcript abundance of *yhjX* was compared between acid stimulated and unstimulated growth conditions and normalized to the *gyrB* housekeeping gene. These analyses revealed a characteristic transcription surge (32) for *yhjX* in acidified wild-type UTI89 cultures, which was not apparent in the isogenic Δ*btsS*Δ*ypdB* (Fig. 1e). To further confirm that *yhjX* is induced by low pH and not just HCl, we utilized the luminescent reporter to monitor *yhjX* induction in UTI89 and Δ*btsS*Δ*ypdB* in the presence of lactic acid, acetic acid, and pyruvic acid (Fig. S2, Data set S1). All organic acids tested resulted in strong *yhjX* induction in UTI89. However, the Δ*btsS*Δ*ypdB* strain did not induce *yhjX* under any of these acidic conditions (Fig. S2 and Data set S1). Notably, addition of HCl, or acetic acid occasionally leads to two peaks in luminescence (Fig. 1d, Fig. S2) in wild-type UTI89, indicative of two activation surges for *yhjX* transcription. This phenomenon is likely connected to BtsS signaling, but why it occurs only in response to HCl or acetic acid is unknown. Together, these data confirm that *yhjX* is induced in low-pH growth conditions and that this induction depends on the presence of BtsS and YpdB.

10.1128/mbio.02963-22.2 FIG S2 *yhjX* induction in response to organic acids. Wild-type UTI89 (black) and the isogenic Δ*btsS*Δ*ypdB* (purple) containing the P*_yhjX_* luminescence reporter fusion were cultured in LB in the absence (filled shapes) or presence (open shapes) of 10 mM HCl (circles), 10 mM acetic acid (squares), 10 mM lactic acid (upside down triangles), or 1 mM pyruvic acid (diamonds). Luminescence and OD_600_ were measured every hour and luminescence normalized to OD_600_ is plotted. Graphs are representative of four biological replicates. Download FIG S2, TIF file, 0.8 MB. Copyright © 2022 Wiebe et al. 2022 Wiebe et al. https://creativecommons.org/licenses/by/4.0/ This content is distributed under the terms of the Creative Commons Attribution 4.0 International license.

### Induction of BtsS-YpdB in response to acid results from pyruvate produced during l-serine deamination.

**FIG 2:**
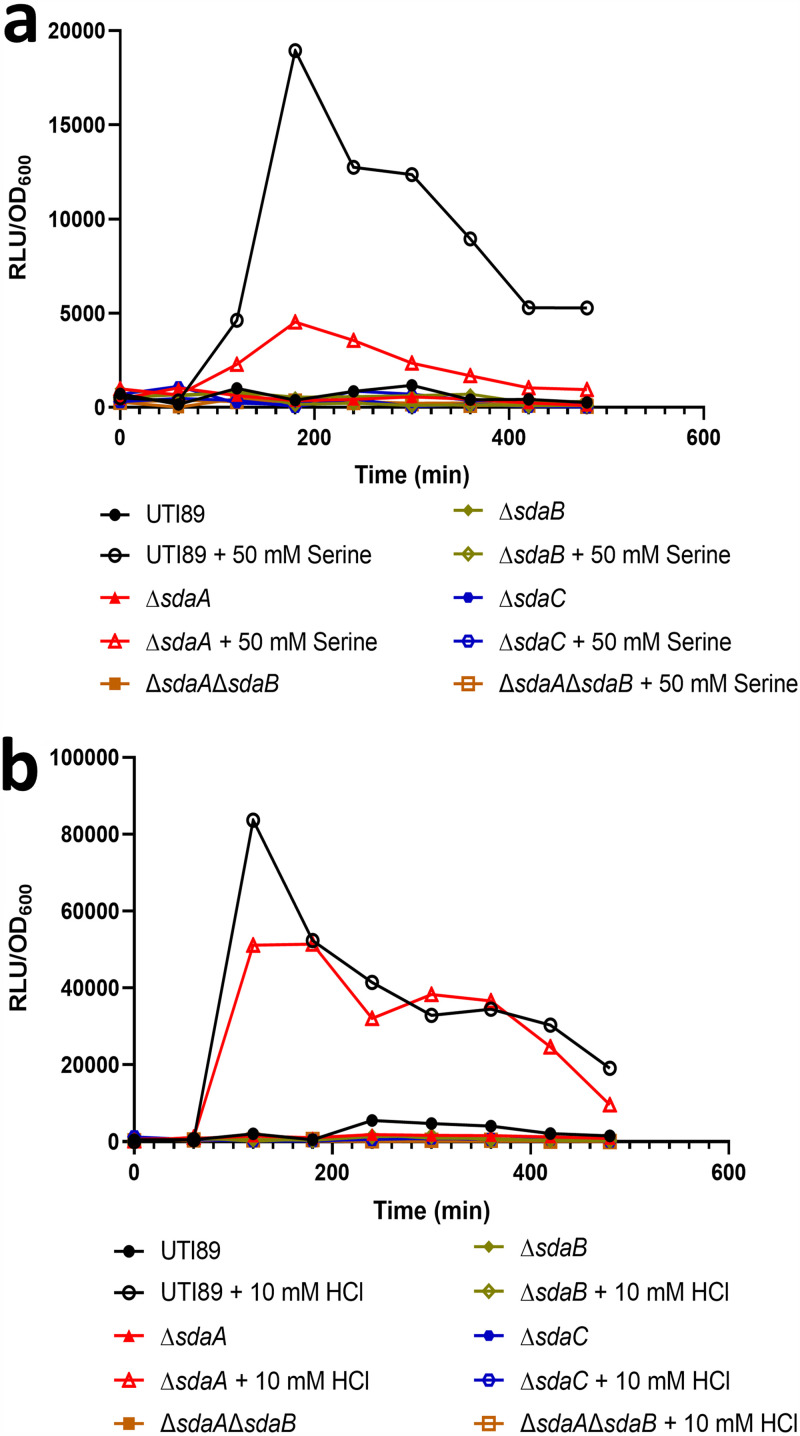
Induction of BtsS-YpdB in response to acid results from pyruvate produced during l-serine deamination. Graphs depict luciferase reporter assay performed over time of strains harboring the *yhjX* promoter reporter during growth in media supplemented with 50 mM serine (a) or 10 mM HCl (b). Deletion of serine import (*sdaC*, blue) or deamination genes (*sdaA*, red; *sdaB*, gold) diminishes *yhjX* promoter activity. Results are representative of 3 biological repeats.

Previous work demonstrated that BtsS signaling is affected by l-serine levels in the growth medium (33). These studies postulated that l-serine, which is the first amino acid to be consumed by E. coli during growth in laboratory media (14), is converted to pyruvate by the l-serine deaminases SdaA and SdaB and could then presumably exported by YhjX to serve as a positive feedback signal for BtsS (15, 34, 35) and (Fig. 1a). Given that the deamination of l-serine also produces ammonia, which can raise intracellular pH (Fig. 1a), we asked whether the mechanism of acid stress alleviation observed in our studies depends on the import and deamination of l-serine. To test this hypothesis, we first created a series of mutants lacking the SdaC transporter (Δ*sdaC*), the SdaA or SdaB deaminases (ΔsdaA, Δ*sdaB*) or both deaminases (Δ*sdaA*Δ*sdaB*). The induction of *yhjX* in these mutants was tested via luciferase reporter assays either in media in which exogenous l-serine was added (Fig. 2a and Data set S1), or in media acidified with HCl (Fig. 2b and Data set 2). Addition of l-serine did not induce *yhjX* promoter activity in any of the *sda* mutants (Fig. 2a and Data set S1). Addition of acid to the media led to decreased *yhjX* induction compared to wild-type UTI89 in all the single mutants tested and led to no *yhjX* induction in the Δ*sdaA*Δ*sdaB* strain (Fig. 2b, Data set S1). Given that pyruvate is the known ligand of the BtsS sensor, our data suggest that *in vivo* it is the deamination of l-serine into pyruvate that actually leads to *yhjX* being induced. As further evidence that it is the generation of pyruvate driving *yhjX* induction, sodium pyruvate was added to the media of the Δ*sdaA*Δ*sdaB* strain, which caused—as expected—*yhjX* induction (Fig. S3 and Data set S1).

10.1128/mbio.02963-22.3 FIG S3 Sodium pyruvate induces the *yhjX* reporter in the absence of SdaA and SdaB. Wild-type UTI89 (black) and the isogenic Δ*sdaA*Δ*sdaB* (brown) strains containing the P*_yhjX_* luminescence reporter fusion were cultured in LB in the absence (filled shapes) or presence (open shapes) of 1 mM sodium pyruvate. Luminescence and OD_600_ were measured every hour and luminescence normalized to OD_600_ is plotted. Graphs are representative of three biological replicates. Download FIG S3, TIF file, 0.7 MB. Copyright © 2022 Wiebe et al. 2022 Wiebe et al. https://creativecommons.org/licenses/by/4.0/ This content is distributed under the terms of the Creative Commons Attribution 4.0 International license.

### l-serine deamination is a mechanism that protects E. coli from acid stress.

**FIG 3:**
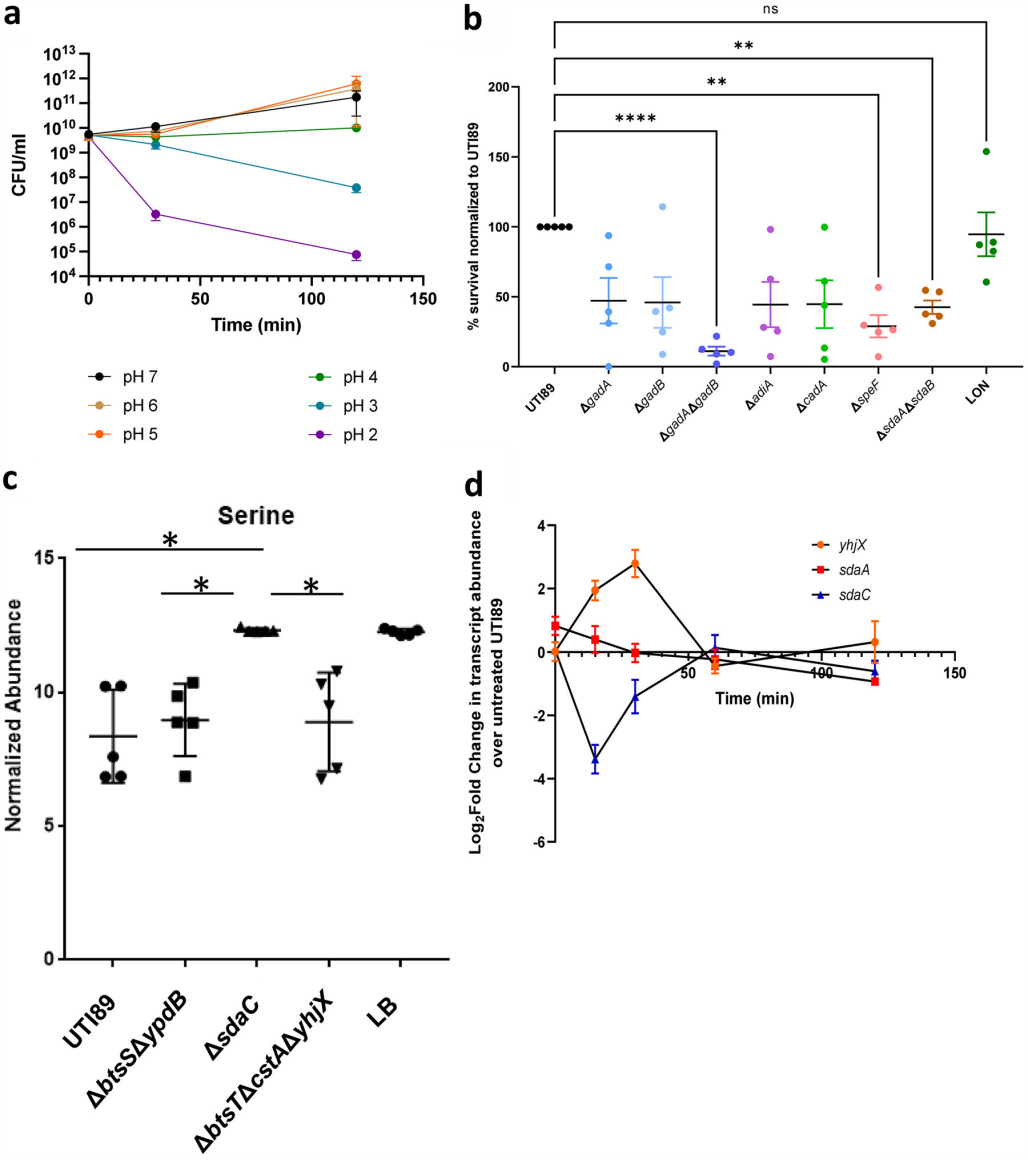
l-serine deamination is another acid resistance mechanism in E. coli. (a) CFU/mL of UTI89 grown in LB adjusted to the indicated pHs with HCl (*n *=* *4 biological replicates). (b) Graph depicts survival in acidic conditions, compared to the wild-type strain, of mutants deleted for decarboxylases or serine deaminases. For these assays, cultures were incubated for 3 h, at which point an aliquot was collected for CFU enumeration before acid treatment. The remaining culture was treated with HCl to adjust the pH to three. Samples were incubated for an additional 30 min, after which they were plated for CFU. Percent survival in acid is calculated as the number of CFU in acid treatment, compared to untreated input control. Statistical analysis was performed by 1-way ANOVA with *post hoc* Dunnett’s multiple comparisons correction test (**, *P* < 0.005; ****, *P* < 0.0001). Error bars indicate SEM of 5 biological replicates. (c) UTI89, Δ*btsS*Δ*ypdB*, and Δ*sdaC* were grown until cultures reached an OD_600_ = 0.5, then 1 M HCl was added to the culture to a final concentration of 10 mM (pH = 5). Cultures were incubated for another 15 min, then 1 mL of culture was collected. Cells were pelleted and supernatant was flash frozen and stored at −80°C prior to sample preparation. Following MS sample preparation, extracellular serine abundance was detected by LC-MS. (d) qPCR analysis of *yhjX* (orange), *sdaA* (red), and *sdaC* (blue) transcript abundance after acid stimulation in wild-type UTI89. The relative fold change was determined by the ΔΔ*C_T_* method where transcript abundances were normalized to *gyrB* housekeeping gene transcripts. Error bars indicate SEM of three biological replicates (*, *P* < 0.005, ANOVA).

If l-serine deamination is a component of the E. coli acid response, we reasoned that the mutant lacking the SdaA/B enzymes would display a survival defect in acidic conditions. We first determined the acid tolerance profile of UTI89, given that we have not previously tested this strain for acid sensitivity. To do this, we subcultured UTI89 in fresh, unbuffered LB (pH 7.4) for 3 h then adjusted the culture pH to 7, 6, 5, 4, 3, or 2 using HCl. We then assessed survival of UTI89 after 30 min or 2 h of exposure. As expected, wild-type UPEC survival did not get substantially affected during 30 min incubation at pH 3 but decreased by 2 logs during the 2 h of incubation (Fig. 3a and S4a). Incubation at pH 2 for 30 min and 2 h both had substantial impact on UPEC growth (Figure 3a and Fig. S4a). To compare the effects of *sda* deletion on acid tolerance, we picked 30-minute incubation at pH 3, since these conditions do not significantly impact survival of wild-type UTI89. Under the selected conditions, we observed the Δ*sdaA*Δ*sdaB* strain exhibiting the most pronounced acid survival defect compared to any of the single Δ*sdaA*, Δ*sdaB*, or Δ*sdaC* mutants (Fig. S5). The acid susceptibility profile of Δ*sdaA*Δ*sdaB* is similar to known acid resistance mutants cultured under the same conditions (Fig. 3b). The survival defect of Δ*sdaA*Δ*sdaB* was also observed when the strain was incubated for 2 h in pH 3, compared to wild-type UTI89 or an isogenic Δ*gadA*Δ*gadB* strain (Fig. S4b; pH 3 data). Intriguingly, when we tested Δ*sdaA*Δ*sdaB* and Δ*gadA*Δ*gadB* in pH 2 for 30 min, we observed a similar decline in CFU that was not statistically different from wild-type UTI89 (Figure S4c; pH 2, 30-minute data). These observations indicate a different susceptibility profile for UPEC, compared to commensal E. coli (36, 37) and demonstrate that l-serine deamination becomes an important acid tolerance mechanism for UPEC.

10.1128/mbio.02963-22.4 FIG S4 a) Graph depicts survival of UTI89 in increasingly acidic conditions. For these assays, cultures were incubated for three hours, at which point an aliquot was collected for CFU enumeration before acid treatment. The remaining culture was adjusted to the indicated pH using HCl. Samples were incubated for 30 minutes and 120 minutes. Percent survival in acid is calculated as the number of CFUs in acid treatment, compared to untreated input control. (*n *=* *4 biological replicates). b) Graph depicts CFU/mL of UTI89, UTI89 Δ*sdaA*Δ*sdaB*, and UTI89 Δ*gadA*Δ*gadB* at t = 0, 30, and 120 minutes after pH of the culture was adjusted to 3 with HCl. c) Graph depicts CFU/mL of UTI89, UTI89 Δ*sdaA*Δ*sdaB*, and UTI89 Δ*gadA*Δ*gadB* at t = 0, 30, and 120 minutes after pH of the culture was adjusted to 2 with HCl. Download FIG S4, TIF file, 0.4 MB. Copyright © 2022 Wiebe et al. 2022 Wiebe et al. https://creativecommons.org/licenses/by/4.0/ This content is distributed under the terms of the Creative Commons Attribution 4.0 International license.

10.1128/mbio.02963-22.5 FIG S5 Acid tolerance profile of serine deaminase and serine import mutants. Graph depicts survival in acidic conditions, compared to the wild-type strain, of mutants deleted serine deaminases (*sdaA* and *sdaB*) or serine importer (*sdaC*). For these assays, cultures were incubated for three hours, at which point an aliquot was collected for CFU enumeration before acid treatment. The remaining culture was treated with HCl to adjust the pH to three. Samples were incubated for an additional 30 minutes, after which they were plated for CFUs. Percent survival in acid is calculated as the number of CFUs in acid treatment, compared to untreated input control. Statistical analysis was performed by 1-way ANOVA with *post hoc* Dunnett’s multiple comparisons correction test (*, *P* < 0.05). Download FIG S5, TIF file, 0.2 MB. Copyright © 2022 Wiebe et al. 2022 Wiebe et al. https://creativecommons.org/licenses/by/4.0/ This content is distributed under the terms of the Creative Commons Attribution 4.0 International license.

To determine if serine is imported by E. coli in response to a drop in pH, cell culture supernatants were then analyzed for relative serine abundance by LC-MS and compared to acidified media alone as a control. Mass spectrometric measurements of serine in media alone demonstrated that baseline serine abundance level can be detected with this method (Fig. 3c and Data set S2). Measurements of serine in acidified media revealed a significant reduction in extracellular serine abundance in the supernatant fractions of WT UTI89, but not the Δ*sdaC* mutant (Fig. 3c and Data set S2). These data indicate that serine is imported—in an SdaC-dependent manner—into the bacterial cell in response to acid stress.

Extracellular serine levels also drop in the Δ*btsS*Δ*ypdB* mutant under acidic conditions (Fig. 3c and Data set S2). This observation is consistent with the notion that l-serine is imported independent of BtsS-YpdB signaling. Subsequent qPCR showed that in the wild-type strain *sdaA* transcript abundance does not significantly change in response to acid stress (Fig. 3d), in sharp contrast to *yhjX* that displays an activation surge (Fig. 3d). These data indicate that *sdaA* transcription is not acid inducible. Interestingly, *sdaBC* transcript abundance sharply drops at 15 min post addition of acid to the media, displaying a fold change that is the opposite of *yhjX* (Fig. 3d). Maurer et al., previously reported sdaB and sdaC as “acid-low” transcripts (37). Our data indicated drop in transcript shortly after addition of acid and coincident to the time that l-serine is brought into the cell (Fig. 3b). This could indicate that acidic conditions lead to downregulation of *sdaBC* via an unknown regulator, or that production of pyruvate or ammonia by l-serine deamination has a negative impact on *sdaBC* transcription.

**FIG 4:**
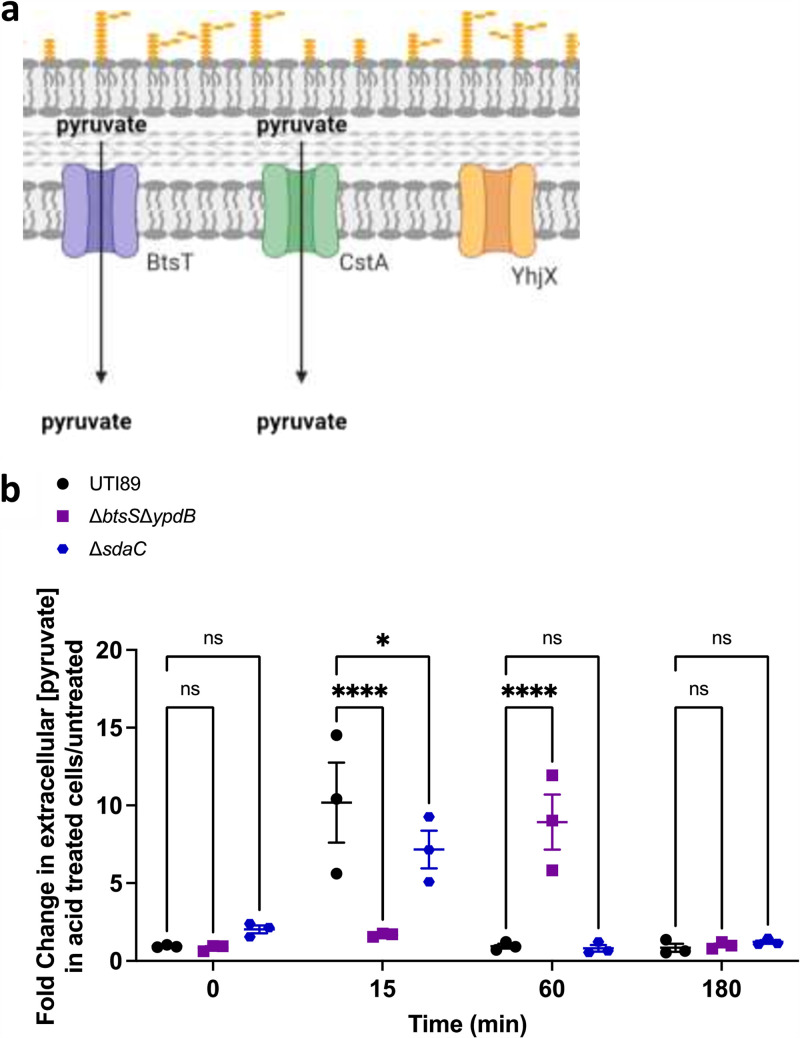
(a) Cartoon depicts currently known pyruvate transporters (BtsT and CstA) and a putative pyruvate transporter (YhjX) in E. coli. Created with BioRender.com. (b) UTI89, Δ*btsS*Δ*ypdB*, and Δ*sdaC* strains were grown until cultures reached an OD_600_ = 0.5, then split so that 1 M HCl was added to half of the culture while the other half continued to grow in LB alone. Supernatant was collected after 0, 15, 60, and 180 min. Pyruvate concentration in the supernatant was quantified for all time points. Fold change in pyruvate concentration in the acid treated samples over the unstimulated samples is graphed. Error bars indicate SEM of 3 biological replicates (****, *P* < 0.001; *, *P* < 0.05).

YhjX is induced in response to pyruvate detection by the BtsS sensor histidine kinase (33). Given that l-serine deamination leads to the production of pyruvate, which is the known ligand for BtsS (33), we hypothesized that SdaA/SdaB-mediated l-serine deamination increases intracellular pyruvate levels that could then be exported via YhjX, if the function of YhjX is to transport pyruvate (Fig. 4a). To determine if extracellular pyruvate abundance changes in response to acid treatment, we measured pyruvate levels in the extracellular milieu over time, following acidification of the culture media, in the same samples used for the serine measurements in Fig. 3c. Cell culture supernatants were analyzed for changes in pyruvate abundance using Pyruvate Assay kits (MAK071-1KT, Sigma-Aldrich) and fold change compared to nontreated controls was determined. In wild-type UTI89, an increase in extracellular pyruvate is observed at 15 min following acidification, compared to the untreated isogenic control (Fig. 4b, Data set S3). Following this pyruvate surge, pyruvate levels are not significantly higher between treated and untreated UTI89 cells at 60 and 180 minutes post acidification. In the mutant lacking the SdaC transporter, extracellular levels are significantly lower than those of wild-type UTI89 at 15 min post acidification, but still increase, suggesting that pyruvate is exported from the cell even when l-serine import is disrupted. In sharp contrast, the supernatant fractions of the Δ*btsS*Δ*ypdB* mutant, which as demonstrated in Fig. 1d, has no observable *yhjX* promoter activity (Fig. 4b), show no increase in extracellular pyruvate levels at 15 min following acidification. Instead, we observe an increase in extracellular pyruvate at 60 min post acidification for Δ*btsS*Δ*ypdB*. These data suggest that YhjX may indeed export pyruvate, although additional studies are needed to prove this biochemically. Our data also indicate that in the absence of YhjX, other transporters can presumably fulfill the function of pyruvate export, since extracellular pyruvate levels do increase at a later time point in the acid-treated Δ*btsS*Δ*ypdB* strain.

In conclusion, our data demonstrate that the import and deamination of l-serine provides protection from acid stress to uropathogenic E. coli, and we provide circumstantial evidence for export of pyruvate via YhjX under acidic conditions.

## DISCUSSION

In this work, we show that l-serine deamination serves as an additional acid response mechanism in E. coli. Serine is the first amino acid consumed by E. coli when grown in complex media, despite its toxicity at higher concentrations (14). l-serine can be deaminated by the SdaA and SdaB enzymes to yield pyruvate and ammonia. *E coli* encodes multiple serine deaminase genes that function under a variety of environmental conditions (38). For instance, while both *sdaA* and *sdaB* are expressed under aerobic conditions, *sdaA* is expressed in nutrient limited minimal media, and in contrast, *sdaB* is expressed in the nutrient rich LB (16, 35, 39). The redundancy of these enzymes indicates that the deamination of serine is important for these bacteria. In this paper, we propose that l-serine import into E. coli serves as an acid tolerance mechanism through the production of ammonia and pyruvate. Maurer et al. profiled gene expression in K-12 E. coli in response to pH changes. Their work showed that serine deaminases were upregulated in basic conditions compared to acidic conditions (37). This is reflected in our qPCR data (Fig. 3d), where we show that *sdaC* is downregulated shortly after acidification. However, we see that sustained growth in acidic media led to an increase in *sdaC* transcript abundance over time (Fig. 3d). It is possible that acidic conditions lead to downregulation of *sdaBC* via an unknown regulator, or that production of pyruvate or ammonia by l-serine deamination exerts negative feedback on *sdaBC* transcription.

Various bacteria are known to use ammonia to neutralize their intracellular pH, including E. coli (11, 40, 41). We postulate that the ammonia produced via l-serine deamination functions as a base which increases the cytoplasmic pH of E. coli when under acid stress. A similar role has been reported for glutamine (AR2_Q) in E. coli (11, 13). Deletion of the serine deaminase genes, *sdaA* and *sdaB*, results in a significant decrease in cell survival in acidic conditions compared to the wild-type strain. This decrease in cell survival is comparable to that observed in other AR mutants of E. coli. All together, these data suggest that l-serine deamination serves as a previously uncharacterized acid tolerance mechanism in E. coli. Loss of serine deaminase activity was previously shown to result in changes to cell shape due to interference with cell wall synthesis (16, 38). These serine deaminase mutants exhibit increased filamentation. In the context of UTI, filamentous UPEC are deficient in invading bladder cells, forming secondary intracellular bacterial communities, and in establishing quiescent intracellular reservoirs (42, 43). Thus, loss of serine deamination could detrimentally affect the ability of UPEC to form reservoirs in addition to its ability to tolerate low pH. Evolution of mechanisms that utilize various amino acids for tolerating acid stress would allow bacteria to seamlessly adapt to different host niches with differing resource availabilities during the infection process.

It has been shown that up to 51% of serine flux is directed to the production of pyruvate (14), which can presumably be shunted into the TCA cycle for energy production. Here, we demonstrate a connection between serine deamination and the pyruvate responsive cross regulating two-component system, BtsS-YpdB. BtsS-YpdB activation occurs in response to increased pyruvate and leads to the upregulation of *yhjX* transcription. Loss of l-serine deaminases result in ablation of *yhjX* promoter induction in response to serine, indicating that pyruvate produced via serine deamination induces activation of the BtsS-YpdB system. Our metabolomics analyses demonstrate that extracellular serine levels drop in the Δ*btsS*Δ*ypdB* mutant under acidic conditions indicating that BtsS-YpdB activation occurs after l-serine is imported. In this work we also show that *yhjX* is also induced by the BtsS-YpdB system in response to acidic pH (Fig. 1b–d). Although this activation in response to low pH was dose dependent, considering the data that suggests *yhjX* is induced through serine deamination and that pyruvate is a known ligand for BtsS, *yhjX* activation in response to low pH appears to be a response to the pyruvate produced by serine deaminases. The function of YhjX remains elusive. We, and others, have hypothesized that YhjX could serve as a pyruvate transporter. Here, we provide preliminary evidence that YhjX may function as a pyruvate transporter, but it is not necessary for the l-serine mediated acid response, as extracellular pyruvate levels do increase – albeit in a delayed fashion – in the Δ*btsS*Δ*ypdB* strain (Fig. 4b).

In summary we show that in acidified media, cells import l-serine from the growth media via the SdaC transporter and deaminate it via the action of SdaA and SdaB. Deletion of both deaminase genes renders E. coli susceptible to acid stress similarly to known AR system deletion mutants. We therefore propose that the importation and deamination of serine represents a previously uncharacterized acid response mechanism in E. coli.

## MATERIALS AND METHODS

### Bacterial strains and growth conditions.

All studies were performed in the well characterized UPEC cystitis isolate UTI89 (20) and derived isogenic deletion mutants. UTI89 is of the sequence type ST95 and is serotyped as O18:K1:H7 (20). For all analyses, strains were propagated from a single colony in unbuffered lysogeny broth (LB) (Fisher Scientific), at pH 7.4. Inoculated strains were grown overnight at 37°C with shaking unless otherwise noted. Strains containing the luciferase reporter plasmid were grown in LB + 50 μg/mL gentamicin at 37°C, 220 rpm overnight. The pH at the beginning of the culture (pH 7.4) and end of the culturing (pH 8.56) was measured using Thermo Scientific Orion Star A211 pH meter with an Orion Green pH Combination Electrode. Specific growth conditions for reporter and survival assays are described in the relevant sections below. Gene deletions were created using the λ-red recombinase system (44). The Δ*btsS*Δ*ypdB* and Δ*yhjX* mutant strains were created in previous studies (27). The *yhjX::lux* reporter, which was previously constructed (28), was introduced into each strain via electroporation and validated by PCR.

A complete list of strains, primers, and plasmids used for in this study can be found in Tables S1 and S2.

10.1128/mbio.02963-22.6 TABLE S1 Primers used in this study. Download Table S1, DOCX file, 0.02 MB. Copyright © 2022 Wiebe et al. 2022 Wiebe et al. https://creativecommons.org/licenses/by/4.0/ This content is distributed under the terms of the Creative Commons Attribution 4.0 International license.

### Luciferase reporter assay.

Overnight cultures were spun at 3220 × *g* for 10 min. Pellets were resuspended in 5 mL 1× PBS and repelleted. Pellets were resuspended in 5 mL 1× PBS and normalized to a starting OD_600_ = 0.05 in 1 mL of the indicated media (LB, LB + 10 mM HCl, or LB + 50 mM MOPS or HEPES buffer + 10 mM HCl). Each suspension was used to seed black, clear bottomed 96-well plates at 200 μL per well from and grown at 37°C with shaking. OD_600_ and luciferase readings were taken every hour for 8 h using a Molecular Devices SpectraMax i3 plate reader. At least 3 biological replicates were assayed.

### Acid tolerance assays.

Acid tolerance assays were performed as follows: Bacteria were grown overnight as described above and diluted 1:100 in 5 mL of fresh, unbuffered LB at pH 7.4. Cultures were incubated at 37°C with shaking. When strains reached mid exponential growth phase (OD_600_ of ~3.0 at 3 h), 1 mL of the culture was centrifuged at 16,000 × *g* for 5 min, washed in 1× PBS, serially diluted and spot-plated for CFU to determine CFU prior to acid exposure (Input sample). The pH of the remaining 4-mL culture was adjusted to a pH of 3 using 5 M HCl, and acidified cultures were incubated at 37°C, with shaking for 30 min. Following incubation, 1 mL of the acidified sample was centrifuged, washed in PBS, and plated for CFU. Percent survival was calculated by dividing the CFU/mL after acid stress by the CFU/mL of the input sample. Statistical analysis was performed by 1-way ANOVA with *post hoc* Dunnett’s multiple comparisons correction test.

### RNA extraction and RT-qPCR.

**Growth conditions for RNA sample collection.** To collect samples for transcriptional analysis, strains were grown aerobically in LB to an OD_600_ = 0.5 and then split into two conditions: continued growth in LB alone, or in LB in which HCl was added to a final concentration of 10 mM. Samples were taken for RNA extraction at time = 0, 15, 30, 60, and 120 min after splitting the culture. All samples were centrifuged at 6,000 × *g* for 7 min at 4°C. The supernatant fractions were decanted, and cell pellets were flash frozen in dry ice and ethanol and stored at −80°C until RNA extraction.

RNA was extracted using the RNeasy minikit from Qiagen, following the manufacturer’s extraction protocol. A total of 3 μg of RNA was DNase treated using 2 units of Turbo DNase I enzyme (Invitrogen). A total of 1 μg of DNase-treated RNA was reverse transcribed using Superscript III reverse transcriptase (Invitrogen/Thermo Fisher). cDNA was amplified in an Applied Biosystems StepOne Plus Real-Time instrument using TaqMan MGB chemistry with primers and probes listed in Table S1. All reactions were performed in triplicate with four different cDNA concentrations (100, 50, 25, or 12.5 ng per reaction). Relative fold difference in transcript abundance was determined using the ΔΔC_T_ method of Pfaffl et al., with a PCR efficiency of >95% (45). Transcripts were normalized to *gyrB* abundance. At least 3 biological replicates were performed for each transcript. Results were statistically analyzed using a two-way ANOVA with Sidak’s multiple-comparison test.

### Metabolomics.

UTI89, Δ*btsS*Δ*ypdB*, and Δ*sdaC* were grown in LB and incubated at 37°C with shaking, until cultures reached an OD_600_ = 0.5. Then, 1 M HCl was added to the culture to a final concentration of 10 mM (pH = 5). Cultures were incubated for another 15 min and then 1 mL of culture was collected. Cells were pelleted and supernatant was flash frozen and stored at −80°C until analyzed via Liquid Chromatography-Mass Spectrometry (LC-MS)-based metabolomics in the Vanderbilt Center for Innovative Technology (CIT). Isotopically labeled phenylalanine-D8 and biotin-D2 were added to 200 μL of culture supernatant per sample, and protein was precipitated by addition of 800 μL of ice-cold methanol followed by overnight incubation at −80°C. Precipitated proteins were pelleted by centrifugation (15,000 rpm, 15 min), and supernatants were dried down *in vacuo* and stored at −80°C. Individual samples were reconstituted in 120 μL of reconstitution buffer (acetonitrile/water, 90:10, vol/vol) containing tryptophan-D3, pyruvate-C13, valine-D8, and inosine-4N15. A quality control (QC) sample was prepared by pooling equal volumes from each individual sample. Quality control samples were used for column conditioning, retention time alignment and to assess mass spectrometry instrument reproducibility throughout the sample set and for individual batch acceptance.

LC-MS and LC-MS/MS analyses were performed on a high-resolution Q-Exactive HF hybrid quadrupole-Orbitrap mass spectrometer (Thermo Fisher Scientific, Bremen, Germany) equipped with a Vanquish UHPLC binary system and autosampler (Thermo Fisher Scientific, Germany). Metabolite extracts were separated on ACQUITY UPLC BEH Amide HILIC 1.7 μm, 2.1 × 100 mm column (Waters Corporation, Milford, MA) held at 30°C. Liquid chromatography was performed at a 200 μL min using solvent A (5 mM Ammonium formate in 90% water, 10% acetonitrile, and 0.1% formic acid) and solvent B (5 mM Ammonium formate in 90% acetonitrile, 10% water, and 0.1% formic acid) with a gradient length of 30 min.

Full MS analyses (6 μL injection volume) were acquired over 70 to 1,050 mass-to-charge ratio (*m/z*) in negative ion mode. Full mass scan was acquired at 120K resolution with a scan rate of 3.5 Hz, automatic gain control (AGC) target of 10e6, and maximum ion injection time of 100 ms. MS/MS spectra were collected at 15K resolution, AGC target of 2e5 ions, and maximum ion injection time of 100 ms.

The acquired raw data were imported, processed, normalized and reviewed using Progenesis QI v.3.0 (Non-linear Dynamics, Newcastle, UK). All MS and MS/MS sample runs were aligned against a QC (pooled) reference run. Unique ions (retention time and *m/z* pairs) were deadducted and deisotoped to generate unique “features” (retention time and *m/z* pairs). Data were normalized to all features using Progenesis QI.

Experimental data for measured serine (i.e., retention time and MS^2^ fragmentation pattern) and pyruvate (i.e., retention time) was consistent with reference standards.

### Pyruvate quantification.

To collect samples for extracellular pyruvate quantification, strains were grown aerobically in LB to an OD_600_ = 0.5 and then split into two conditions: continued growth in LB alone, or in LB in which HCl was added to a final concentration of 10 mM. Samples were taken at time = 0, 15, 60, and 180 min after splitting the culture and normalized to an OD_600_ = 1.0. Samples were then centrifuged, and cell pellets and supernatant were separated, flash frozen, and stored at −80°C until assay was performed. Pyruvate Assay kits (MAK071-1KT, Sigma-Aldrich) were used to quantify pyruvate in the supernatant fraction according to the manufacturer’s protocol. Fold change in pyruvate concentration was calculated by dividing the concentration of pyruvate in cells grown in acidic conditions over the concentration of pyruvate in cells grown in LB alone for each time point. Data were analyzed using 2-way ANOVA with a Dunnett’s *post hoc* multiple-comparison test to compare the mutants to WT at each time point.

### Statistical analysis.

Statistical analyses were performed in GraphPad Prism, using the most appropriate test as indicated in the sections above and in the results. Details of sample size, test used, error bars, and statistical significance cutoffs are presented in the text or figure legends. All experiments were performed in at least three biological replicates. Representative graphs are shown for the luminescence reporter assays. qPCR data were analyzed using 2-way ANOVA with a Sidak’s multiple-comparison test to compare individual time points. LC-MS abundance values were plotted as ArcSinh normalized values.

### Graphics.

All graphical models and drawings were generated using BioRender.com.

10.1128/mbio.02963-22.7 TABLE S2 Strains and plasmids used in this study. Download Table S2, DOCX file, 0.01 MB. Copyright © 2022 Wiebe et al. 2022 Wiebe et al. https://creativecommons.org/licenses/by/4.0/ This content is distributed under the terms of the Creative Commons Attribution 4.0 International license.

10.1128/mbio.02963-22.8 DATA SET S1 Raw luminescence data associated with Fig. 1 and 2, S2 and S3. This dataset contains all raw data from all biological replicates performed for luminescence assays depicted in Fig. 1 and 2, S2 and S3. Download Data Set S1, XLSX file, 0.1 MB. Copyright © 2022 Wiebe et al. 2022 Wiebe et al. https://creativecommons.org/licenses/by/4.0/ This content is distributed under the terms of the Creative Commons Attribution 4.0 International license.

10.1128/mbio.02963-22.9 DATA SET S2 Metabolomics data output associated with Fig. 3. This dataset contains all metabolomics data obtained with the analyses for Fig. 3. Download Data Set S2, XLSX file, 18.7 MB. Copyright © 2022 Wiebe et al. 2022 Wiebe et al. https://creativecommons.org/licenses/by/4.0/ This content is distributed under the terms of the Creative Commons Attribution 4.0 International license.

10.1128/mbio.02963-22.10 DATA SET S3 Pyruvate quantification data associated with Fig. 4. This dataset contains all the raw data outputs associated with Fig. 4. Download Data Set S3, XLSX file, 0.1 MB. Copyright © 2022 Wiebe et al. 2022 Wiebe et al. https://creativecommons.org/licenses/by/4.0/ This content is distributed under the terms of the Creative Commons Attribution 4.0 International license.
