# Supplementary figures and images for: Serine Deamination Is a New Acid Tolerance Mechanism Observed in Uropathogenic Escherichia coli
Source: mBio. 2022 Dec 5;13(6):e02963-22. doi: 10.1128/mbio.02963-22 (PMC9765748; doi:10.1128/mbio.02963-22)

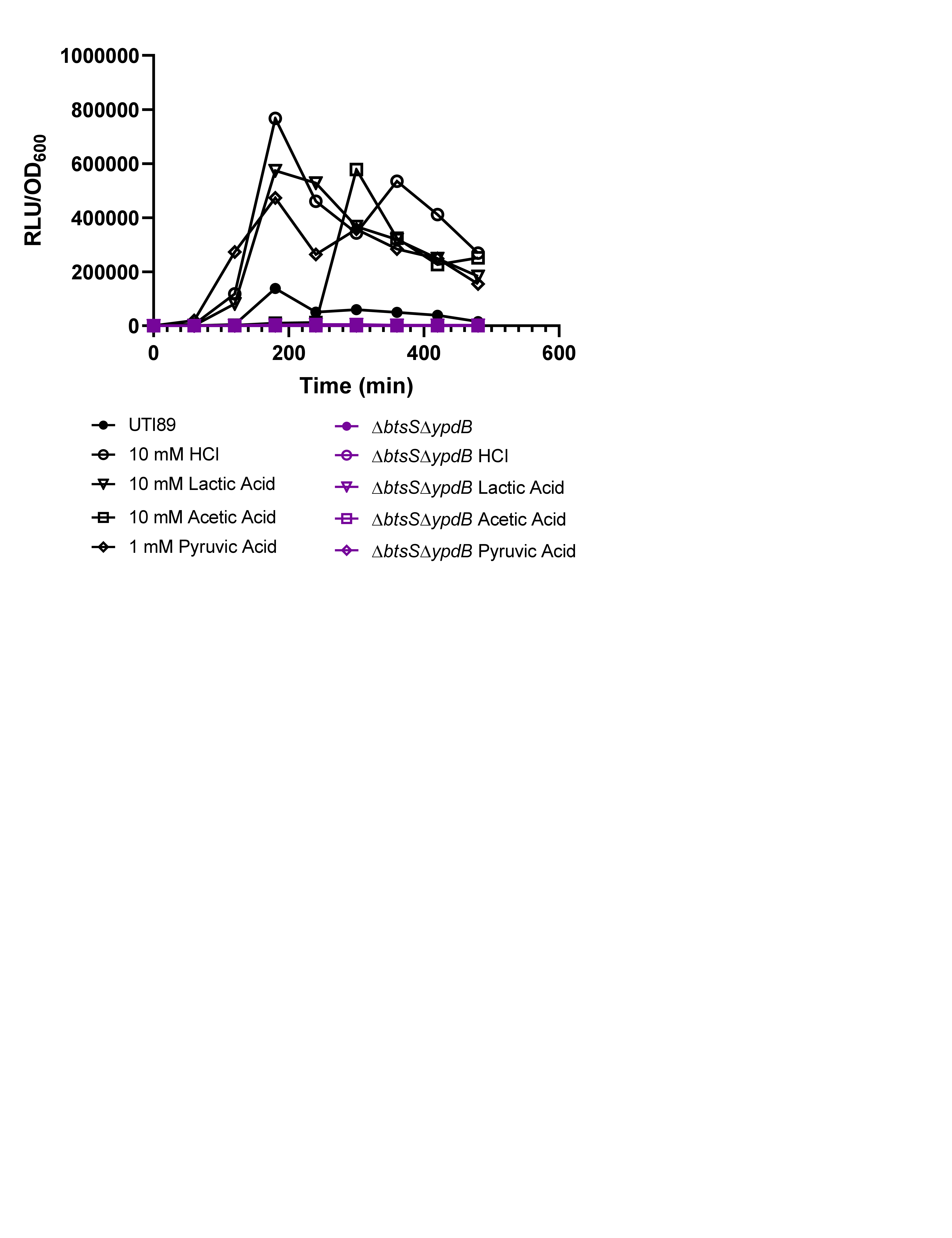

Supplement: FIG S2 [file mbio.02963-22-s0002.tif]

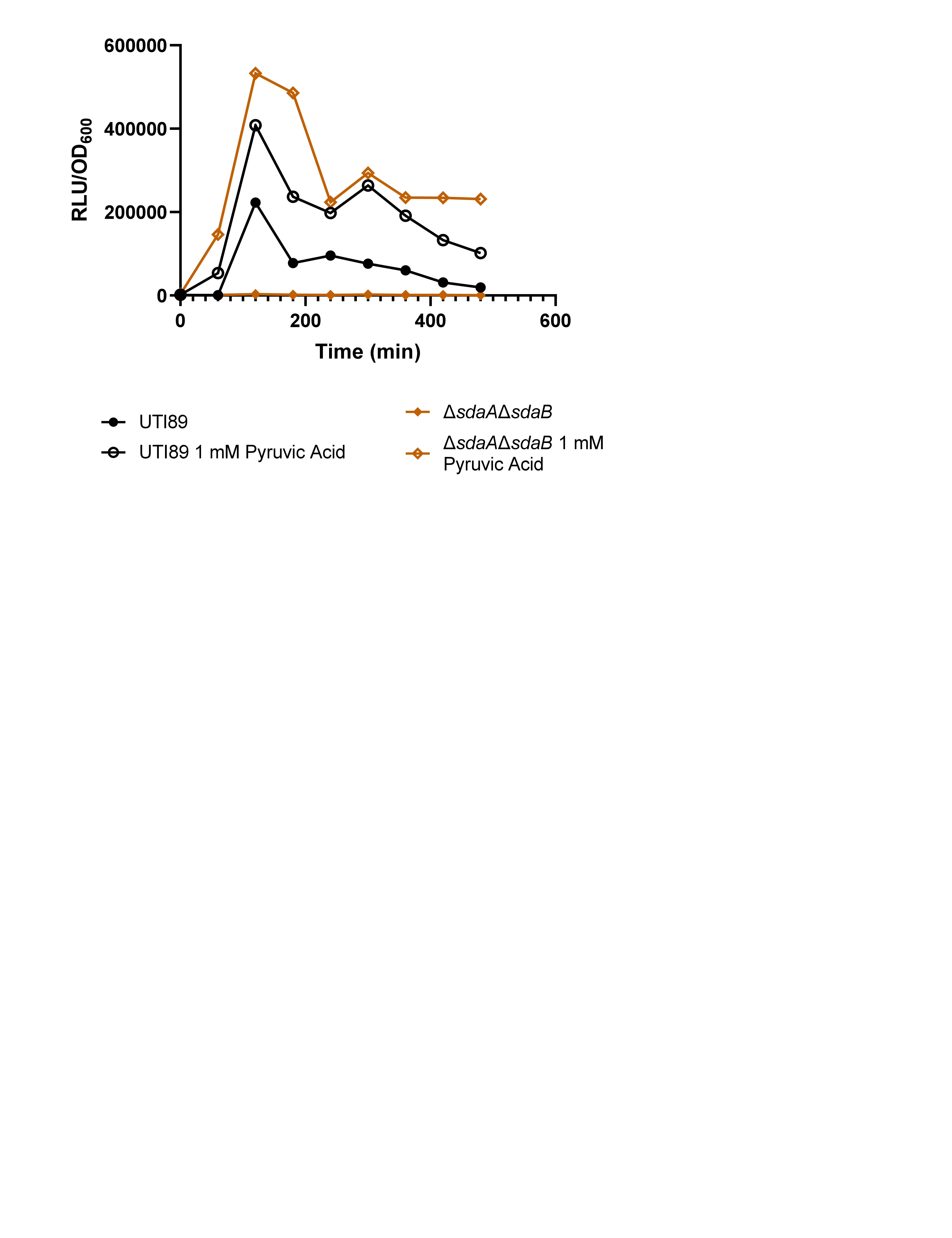

Supplement: FIG S3 [file mbio.02963-22-s0003.tif]

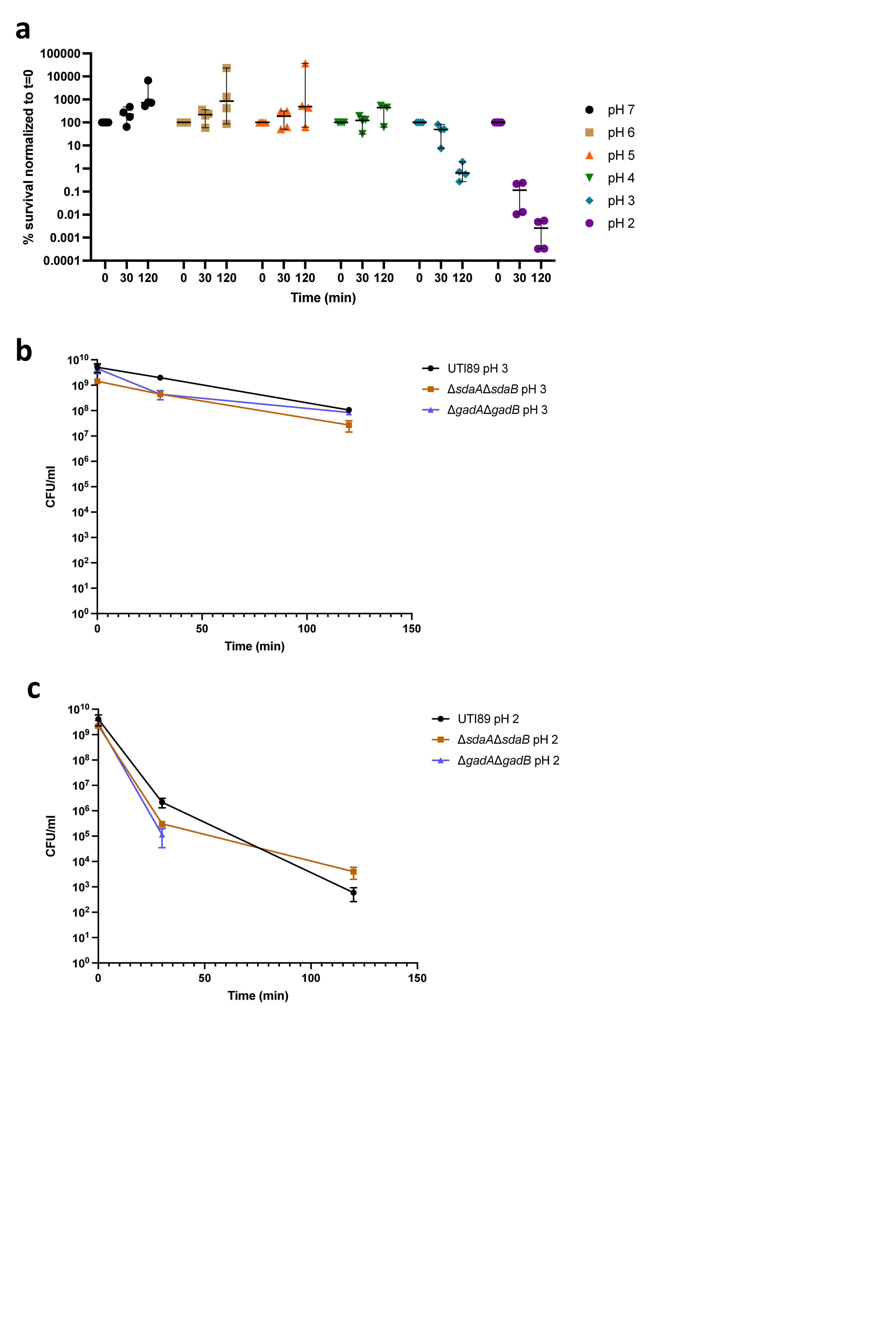

Supplement: FIG S4 [file mbio.02963-22-s0004.tif]

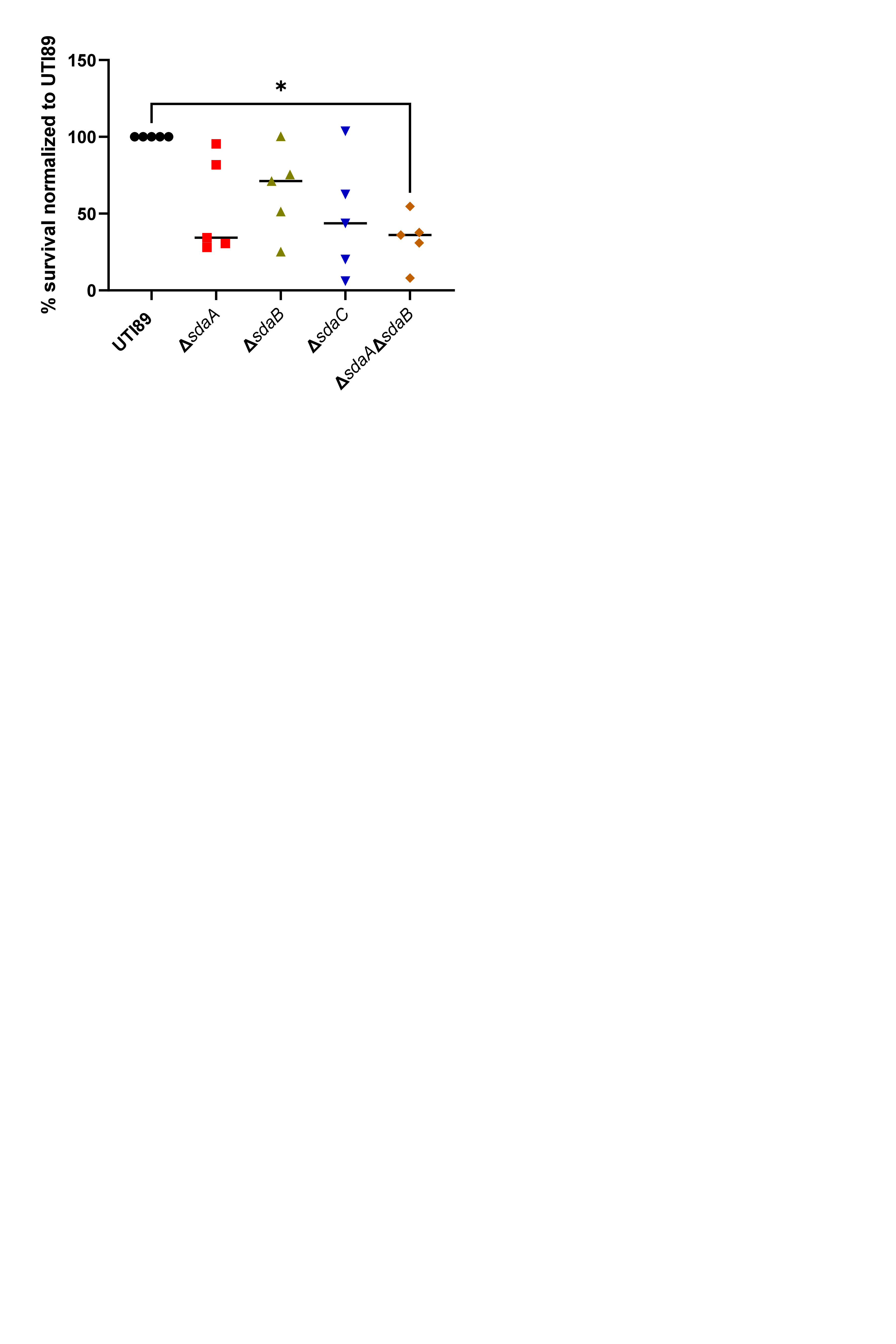

Supplement: FIG S5 [file mbio.02963-22-s0005.tif]
