# Supplementary material for: Serine Deamination Is a New Acid Tolerance Mechanism Observed in Uropathogenic Escherichia coli
Source: mBio. 2022 Dec 5;13(6):e02963-22. doi: 10.1128/mbio.02963-22 (PMC9765748; doi:10.1128/mbio.02963-22)
Supplement: TABLE S1 [file mbio.02963-22-s0006.docx]

**Table S1: Primers used in this study**

| **Primer name** | **Sequence 5’-3’** | **Purpose** |
| --- | --- | --- |
| btsS_KO_f | GGCCTGTTCGCTGTCGCGGCGGATCACCGCTTTAATGGTGTTAAGCGCGTGTAGGCTGGAGCTGCTTC | Deletion of *btsS* |
| btsS_KO_r | GTGTGGTTTGCGGGTATGTACGATTTTAATCTGGTGTTGCTGCTGCTTCACATATGAATATCCTCCTTAG | Deletion of *btsS* |
| btsS_KO_test_f | GATAAGCGTTCACATGTTCAATTTCGTC | Validation of *btsS* deletion |
| btsS_KO_test_r | GCAAGAGTTCAAAGAAAGTTAAACGCAAG | Validation of *btsS* deletion |
| ypdB_KO_f | CAGGAACTGAGCTGGCTAATTAAAGAGCACAGCCAGATGGAGATTGTCGGCGTGTAGGCTGGAGCTGCTTC | Deletion of *ypdB* |
| ypdB_KO_r | TTACAGATGCATTAACTGGCGGAATTCTTTAACTTTGCTACGGCTGACCGCATATGAATATCCTCCTTAG | Deletion of *ypdB* |
| ypdB_KO_test_f | GCCGGAGTGATATTGTGAAAGTCAT | Validation of *ypdB* deletion |
| ypdB_KO_test_r | AATTGTTGATCGGCGGGCAAGC | Validation of *ypdB* deletion |
| sdaA_KO_f | GTTATTAGTTCGTTACTGGAAGTCCAGTCACCTTGTCAGGAGTATTATCGTGTAGGCTGGAGCTGCTTC | Deletion of *sdaA* |
| sdaA_KO_r | AAGCGGGAATAAATTCGCCCATCCGTTGCAGATGGGCGAATAAGAAGATCATATGAATATCCTCCTTAG | Deletion of *sdaA* |
| sdaA_KO_test_f | CATCTGGGTCGTTATCATCCT | Validation of *sdaA* deletion |
| sdaA_KO_test_r | GTAACGAGTGCGCAAATCG | Validation of *sdaA* deletion |
| sdaB_KO_f | CGCGCCGCTTTCGGGCGGCGCTTCCTCCGTTTTAACGCGATGTATTTCCTGTGTAGGCTGGAGCTGCTTC | Deletion of *sdaB* |
| sdaB_KO_r | GGATGAGAAATCGGGAAGAGGCCTCGCAAAAAGAGGCCTCTGGAGAGCGACATATGAATATCCTCCTTAG | Deletion of *sdaB* |
| sdaB_KO_test_f | GTTCCTGATGCCGATGTAC | Validation of *sdaB* deletion |
| sdaB_KO_test_r | CCAGAACAGGCTATGGCT | Validation of *sdaB* deletion |
| sdaC_KO_f | GGCTGAACTGGCTAAAAGCTGAATTATTTGCATTCCTCCAGGAGAAATAGGTGTAGGCTGGAGCTGCTTC | Deletion of *sdaC* |
| sdaC_KO_r | ACATCGCGTTAAAACGGAGGAAGCGCCGCCCGAAAGCGGCGCGAAAGGACCATATGAATATCCTCCTTAG | Deletion of *sdaC* |
| sdaC_KO_test_f | CATCGCCGATAGACAGAT | Validation of *sdaC* deletion |
| sdaC_KO_test_r | GAACTCCACTTCATGCTGAC | Validation of *sdaC* deletion |
| gadB_KO_FOR | CAGGTGTGTTTAAAGCTGTTCTGCTGGGCAATACCCTGCAGTTTCGGGTGTGTAGGCTGGAGCTGCTTC | Deletion of *gadB* |
| gadB_KO_REV | CAAGTAACGGATTTAAGGTCGGAACTACTCGATTCACGTTTTGGTGCGAACATATGAATATCCTCCTTAG | Deletion of *gadB* |
| gadB_KO_Test_FOR | GTGAACAGACTTTGGAAATTGTCCC | Validation of *gadB* deletion |
| gadB_KO_Test_REV | ACTTGCTTACTTTATCGATAAATCCTA | Validation of *gadB* deletion |
| gadA_KO_FOR | GTTTAAAGCTGTTCTGCTGGGCAATACCCTGCAGTTTCGGGTGGTCGCTGGTGTAGGCTGGAGCTGCTTC | Deletion of *gadA* |
| gadA_KO_REV | AAATGGACCAGAAGCTGTTAACGGATTTCCGCTCAGAACTACTCGATTCACATATGAATATCCTCCTTAG | Deletion of *gadA* |
| gadA_KO_Test_FOR | CAATTAATAAGTAGCCGAATACCCACC | Validation of *gadA* deletion |
| gadA_KO_Test_REV | TGTAATACCTTGCTTCCATTGCG | Validation of *gadA* deletion |
| adiA_ko_f | ATGATGAAAGTATTAATTGTTGAAAGCGAGTTTCTCCATCAAGACACCTGGTGTAGGCTGGAGCTGCTTC | Deletion of *adiA* |
| adiA_ko_r | TTACGCTTTCACACACATAACGTGGTAAATACCGTCAATAATTTCTGTCCCTTCCATATGAATATCCTCCTTAG | Deletion of *adiA* |
| adiA_kotest_f | GAAGATACTTGCCCGCAAC | Validation of *adiA* deletion |
| adiA_kotest_r | CTCGCTAAAGCGAAGCGATAC | Validation of *adiA* deletion |
| cadA_ko_f | ATGACTATGAACGTTATTGCAATATTGAATCACATGGGGGTTTATTTTAAAGAAGGTGTAGGCTGGAGCTGCTTC | Deletion of *cadA* |
| cadA_ko_r | TTATTTTTTGCTTTCTTCTTTCAATACCTTAACGGTATAGCGGCCATCAGCATATGAATATCCTCCTTAG | Deletion of *cadA* |
| cadA_kotest_f | GTACCTTCATCGTCAGCCTG | Validation of *cadA* deletion |
| cadA_kotest_r | GTGTTCTCCTTATGAGC | Validation of *cadA* deletion |
| speF_ko_f | ATGACGAGTATAGCCAGTTACCGGGCTGGTCTGGGTTATTGCATCTGCGTGTAGGCTGGAGCTGCTTC | Deletion of *speF* |
| speF_ko_r | AATTTTTCCCCTTTCAACAGGGCGCTTTGCGCATCACGAGGCTTGATGACCATATGAATATCCTCCTTAG | Deletion of *speF* |
| speF_kotest_f | GGTGCTCATATACTGCTAAC | Validation of *speF* deletion |
| speF_kotest_r | GTTGACCATCGTCAGTATG | Validation of *speF* deletion |
| gyrB_qPCR_f | GATGCGCGTGAAGGCCTGATTG | qPCR housekeeping gene |
| gyrB_qPCR_r | CACGGGCACGGGCAGCATC | qPCR housekeeping gene |
| gyrB_qPCR_probe | VIC-ACGAACTGCTGGCGGA-MGBNFQ | qPCR housekeeping gene |
| yhjX_qPCR_f | TCGCTACACCAATCACATACAGAC | *yhjX* qPCR |
| yhjX_qPCR_r | GAAGCAGGAAGTGAAAACCAGC | *yhjX* qPCR |
| yhjX_qPCR_probe | FAM-GCATCGACTCTGCCAGGGTGTAGTC-MGBNFQ | *yhjX* qPCR |
| sdaA_qPCR_f | TGCAAATCCACGCCTATAACG | *sdaA* qPCR |
| sdaA_qPCR_r | CAGTACGCGAGCAGTTCG | *sdaA* qPCR |
| sdaA_qPCR_probe | FAM-CGAAGTGAGCGTGCCGTATCCG-MGBNFQ | *sdaA* qPCR |
| sdaC_qPCR_f | GATGCTGCTGGCTCTGTACC | *sdaC* qPCR |
| sdaC_qPCR_r | ATGATCGGAGAGTGGTTGAACG | *sdaC* qPCR |
| sdaC_qPCR_probe | NED-GCTGTCTCTGGACACTGCATCTG-MGBNFQ | *sdaC* qPCR |
