# Supplementary material for: Serine Deamination Is a New Acid Tolerance Mechanism Observed in Uropathogenic Escherichia coli
Source: mBio. 2022 Dec 5;13(6):e02963-22. doi: 10.1128/mbio.02963-22 (PMC9765748; doi:10.1128/mbio.02963-22)
Supplement: TABLE S2 [file mbio.02963-22-s0007.docx]

**Table S2: Strains and plasmids in this study**

| *E. coli* strain | Relevant genotype | plasmid | Plasmid description |
| --- | --- | --- | --- |
| UTI89 |  |  |  |
| Δ*btsS*Δ*ypdB* | Δ*btsS*Δ*ypdB* |  |  |
| Δ*sdaA* | Δ*sdaA* |  |  |
| Δ*sdaB* | Δ*sdaB* |  |  |
| Δ*sdaC* | Δ*sdaC* |  |  |
| Δ*sdaA*Δ*sdaB* | Δ*sdaA*Δ*sdaB* |  |  |
| Δ*gadA* | Δ*gadA* |  |  |
| Δ*gadB* | Δ*gadB* |  |  |
| Δ*gadA*Δ*gadB* | Δ*gadA*Δ*gadB* |  |  |
| Δ*adiA* | Δ*adiA* |  |  |
| Δ*cadA* | Δ*cadA* |  |  |
| Δ*speF* | Δ*speF* |  |  |
| UTI89 |  | pBBR *yhjX-lux* | PyhjX -264/+36 cloned in the BamHI and EcoRI sites of pBBR1-MCS5-TTRBS-lux; Gm^r 24^ |
